# Immunoglobulin E (IgE)-Mediated Food Allergy in Children: Epidemiology, Pathogenesis, Diagnosis, Prevention, and Management

**DOI:** 10.3390/medicina56030111

**Published:** 2020-03-04

**Authors:** Simona Barni, Giulia Liccioli, Lucrezia Sarti, Mattia Giovannini, Elio Novembre, Francesca Mori

**Affiliations:** Allergy Unit, Department of Pediatrics, Meyer Children’s University Hospital, 50139 Florence, Italy; giulia.liccioli@meyer.it (G.L.); lucrezia.sarti@gmail.com (L.S.); mattia.giovannini@unifi.it (M.G.); elio.novembre@unifi.it (E.N.); francesca.mori@meyer.it (F.M.)

**Keywords:** children, diagnosis, epidemiology, food allergy management, pathogenesis, prevention

## Abstract

A food allergy is an immunoglobulin E (IgE)-mediated hypersensitive reaction to food, which consists in the appearance of allergic symptoms; it can vary from common urticaria to even fatal anaphylaxis. The prevalence of food allergies has been increasing in the past twenty years and it represents a major public health problem in industrialized countries. The mechanism that leads to food allergies is the lack of immunologic and clinical tolerance to food allergens. The diagnosis of IgE-mediated food allergies is based on the combined use of a detailed medical history, in-vivo, and in-vitro research of specific IgE, the elimination diet, and the double-blind placebo-controlled food challenge. The only currently available treatment for allergies is the strict elimination diet. This type of attitude, which we could define as “passive”, does not overcome the risk of accidental reactions due to involuntary intake of the culprit food. For food allergy management, an “active” approach is urgently needed, such as specific allergen immunotherapy, which is currently under development and only used for research purposes. This article aims to give an updated review of IgE-mediated food allergies in pediatric populations in terms of epidemiology, pathogenesis, prevention, diagnosis, and management.

## 1. Introduction

A food allergy is a hypersensitivity response to a specific food antigen. It is classified in immunoglobulin E (IgE) and non-IgE-mediated food allergies on the basis of the time elapsed from the food ingestion to the onset of clinical manifestation, within or later than 2 h, respectively [1].

The aim of this manuscript is to give an updated review of IgE-mediated food allergies in children, in terms of epidemiology, pathogenesis, prevention, diagnosis, and management. As the topic is broad and complex, the review will give an overview of the main concepts of food allergies in children. In particular, chapter 5, entitled “Management” has relevance, primarily for pediatricians and allergists situated in the European community. 

The bibliographic research was performed in November 2019 and it was limited to papers published in the last 10 years in English. Other articles have been identified through bibliographies of relevant articles. The relevant articles were identified using the MEDLINE database with the PubMed search engine, using key terms related to food allergies: “children”, “IgE-mediated food allergy”, “IgE-mediated food hypersensitivity”, “epidemiology”,” pathogenesis”, “prevention”, “diagnosis”, “treatment”, and “immunotherapy”. To avoid excluding important studies, the research was not restricted by type of publication or study design.

## 2. Epidemiology

The prevalence of food allergies has increased in the last two to three decades and represents a public health problem, especially in industrialized countries [2].

In Europe [3] and the US, [4] between 6% and 8% of children suffer from food allergies, respectively.

The exact prevalence of food allergies in a population is difficult to determine; in fact, the gold standard for diagnosis is the double-blind placebo-controlled food challenge (DBPCFC), which is not free of risk for the patient, and can only be performed in specialized centers [2].

For this reason, most of the studies are based on self-reported evidence or parent reports. It is clear, and already known, that this has led to an overestimation of food allergy prevalence. In fact, very often, the surveys are based on questionnaires that do not differentiate between IgE- and non- IgE-mediated food allergies, but are based on reported symptoms that are not confirmed by a DBPCFC. [5,6].

Any food can potentially trigger an allergic reaction. The ones most commonly responsible for food allergies are milk, egg, peanut, tree nuts, shellfish, and fish. For this reason, the review will focus on the prevalence of allergies from the above-mentioned foods [7].

In literature, few studies performing DBPCFC to confirm the existence of a food allergy exist. These include the EuroPrevall study [8] and the HealthNuts study [9]. The EuroPrevall study enrolled 12,049 children from nine European countries and 9336 were followed up until 2 years of age. The overall incidence of cow’s milk allergy was equal to 0.54% (95% CI 0.41–0.70). In the Netherlands and in the UK, the incidence was 1%, whereas in Lithuania, Germany, and Greece, it was less than 0.3% [10]. The average incidence of hen’s egg allergies was 1.23% in children under 2 years of age; the incidences varied from 2% in the UK, to less than 0.1% in Greece [11].

The HealthNuts study recruited 5276 one-year-old children from Australia. It reported a prevalence of 11% of food allergies demonstrated with the oral food challenge evaluating three foods: peanuts (3%; CI, 2.4% to 3.8%), raw eggs (8.9%; 95% CI, 7.8% to 10.0%), and sesame (0.8%; 95% CI, 0.5% to 1.1%) [9]. In the follow-up analysis at 4 years of age, the allergy rate was 3.8%, with a prevalence of peanut and egg allergies, and sesame, equal to 1.9% (95% CI, 1.6% to 2.3%), 1.2% (95% CI, 0.9% to 1.6%), and 0.4% (95% CI, 0.3% to 0.6%), respectively [12].

Studies from Europe and the US on the prevalence of tree nut allergies [13], including systematic reviews and meta-analyses, reported a prevalence rate of less than 2% using the oral food challenge to confirm allergies; hazelnut was the most frequent tree-nut allergy in Europe, whereas in the US, walnut and cashew were the most common triggers of tree-nut allergies.

A systematic review on the prevalence of fish and shellfish allergies, which included 61 studies, showed that fish allergies varied from 0% to 7% and shellfish allergies from 0% to 10.3% [14].

Understanding how many children are affected by food allergies—and which ones are most at risk of developing them—could give clues to both genetic and environmental factors that cause food allergies and, therefore, which preventive measures could be applied to reduce the increase of them.

## 3. Pathogenesis

The immune system plays a central role in the development—or non-development—of a food allergy.

It is essential that the immune system recognize the food antigen as non-pathogenic in order to establish clinical and immunological tolerance. The lack of immunologic and clinical tolerance to food allergens leads to food allergies. This means that, in healthy individuals, there is a normal state of unresponsiveness to food antigens, whereas in patients with food allergies, the sensitization to common food allergens consists in an exaggerated inflammatory response of the immune system [15].

The passage of food antigens through the intestinal epithelium to access the mucosal antigen-presenting cells occurs through two transport systems: passive and active. The passive system is also called paracellular diffusion, in which the food antigen passes between two adjacent enterocytes. The active system, conversely, can take place through the microfold (M) cells, the goblet cells, specialized macrophages that express CX_3_C chemokines receptors 1 on their surface (CX_3_CR1), or through the CD103^+^ dendritic cells in the lamina propria [16,17].

Under normal conditions, these cells induce regulatory T cells (Treg) through the production of IL-10 by macrophages or through the production of transforming growth factor-β (TGF-β) by dendritic cells [18,19].

The tolerance to food antigens breaks down, in some situations, for example in the event of exposure to certain pathogen-associated molecular patterns (PAMPs) or following epithelial damage, leading to the production of IL-25, IL-33, and thymic stromal lymphopoietin (TSLP). Under these conditions, the induction of Treg cells is altered and switched to antigen specific Th2 cells, which, by producing IL-4, stimulate the B cells to produce immunoglobulin E (IgE) and stimulate mast cell expansion. IL-4 also suppresses the tolerogenic function of Treg and reprograms Treg to produce IL-4 themselves, transforming them from tolerogenic cells to pathogenetic ones [2,20]. Type 2 innate lymphoid cells (ILC2s), which are Th2-like cells without antigenic specificity, produce IL-4 and IL-13, blocking the Treg function [21].

IgEs bind the receptors present on the surface of mast cells. When the patient is exposed again to the same food antigen, it binds specific IgE attached to FcεR on mast cell and basophil cell surfaces with degranulation of those cells that release mediators, such as histamine, generating the symptoms of IgE-mediated food allergy reactions [22].

Understanding the pathogenic mechanism underlying IgE-mediated food allergies allows implementation of those measures aimed to restore clinical and immunologic tolerance.

## 4. Diagnosis

**Figure 1:**
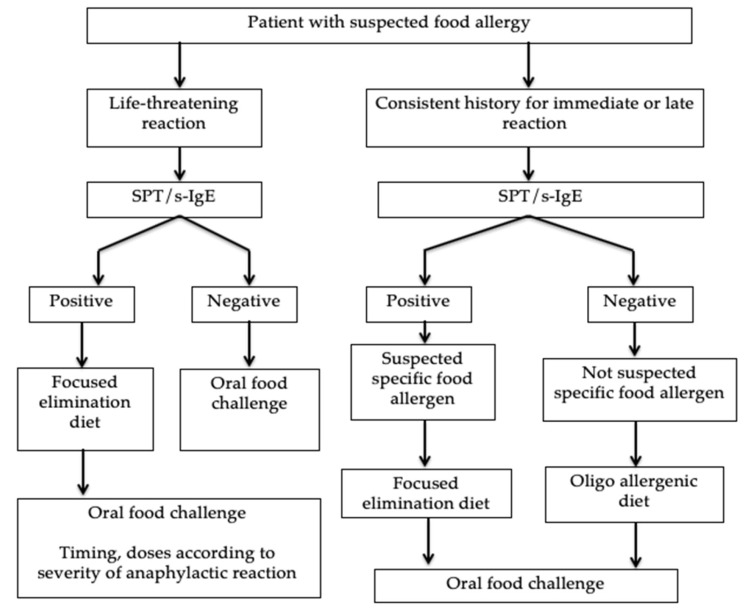
Algorithm for the diagnosis of food allergy (from Muraro A. et al. [23]).

An accurate diagnosis of IgE-mediated food allergies is crucial. It is based on the combined use of a detailed medical history, the research of specific IgE via testing in-vivo (skin prick test, SPT) and in-vitro (specific IgE, s-IgE), the elimination diet, and the subsequent oral food challenge (OFC) Figure 1 [23].

A detailed medical history is very important and some questions are fundamental for clarifying the symptoms: (1) “What symptoms appeared?”; (2) “What foods are deemed responsible and how many times have they caused reactions?”; (3) “What was the amount of food that triggered the reaction?”; (4) “Was the food cooked or raw?”; (5) “What was the latency between food intake and the onset of symptoms and how long did the reaction last?”; (6) “Was food taken regularly before causing the reaction?”; (7) “Should other factors be taken into consideration such as exercise, infections, the simultaneous intake of certain drugs like aspirin or other NSAIDs, or alcohol?”; (8) “Were drugs administered to treat the reaction, and if so, to what effect?” [23].

After having recorded the medical history, the in-vivo and in-vitro estimation of specific IgE is recommended. The SPT is an easy, fast, cheap, and sensitive method for the diagnosis an IgE-mediated food allergies, although its diagnostic value is limited compared to the DBPCFC [24].

Many studies [25,26,27,28,29] have tried to identify the real diagnostic value of the SPT, and it has clearly emerged that a negative test excludes a food allergy by 90%, whereas a positive test does not confirm the diagnosis, but instead, a state of sensitization. As the likelihood of being allergic increases as the wheal diameter of the SPT increases, diagnostic decision levels have been defined in different studies for the common allergens, usually with a cut-off at 95%–100% positive predictive value (PPV), and they can increase the specificity of the SPT (Table 1).

In vitro tests measuring s-IgE is another way to investigate IgE-mediated food allergies. As with SPT, there is a certain correlation between the specific s-IgE concentration and the possibility of a clinical reaction to the specific food. Various studies have attempted to identify precise diagnostic predictive values. In general, it can be stated that undetectable values of s-IgE are associated to a low risk (10–25%) of reaction to the food, while the risk increases with a rise in the levels of s-IgE. The values also vary, according to the type of allergen, Table 1 [25].

When applying this data in clinical practice, we must bear in mind that the reported predictive values refer to the specific populations studied, which may differ from the populations to which these predictive tests need to be applied. Therefore, a certain amount of caution is always necessary with predictive clinical risks [30].

Determination of specific IgE to a single allergen, known as Component-Resolved-Diagnosis (CRD), is a recent advance in the diagnosis of food allergy. For each individual allergen, various components were identified and given a specific name consisting of the first three letters of the genus, the first letter of the species, and a number that usually reflects the chronological order of identification [31]. For example, ‘Cor a 1’ is the first allergen described in hazelnut, the scientific name of which is *Corylus avellana*. So far, for hazelnut, 10 components, named Cor a 1-2-8-9-10-11-12-13-14, TLP (thaumatin like protein) have been identified [32]. The awareness of some of these components, such as Cor a 14, has been associated with a greater risk of serious reactions, as also reported for other allergenic molecules of other substances [33].

However, the clinical repercussions of this information have not yet been defined, and it should also be remembered that not all the molecules present in the allergens are currently available for laboratory testing, as many are still to be discovered.

The Basophil Activation Test (BAT) is another test for food allergies that is moving progressively from the laboratory to a useful tool in clinical practice [34]. BAT can potentially be considered as in-vitro OFC, where basophil cells of the patient are exposed to the culprit food extract in a test tube [35]. The BAT is based on flow cytometry where the expression of activation markers is measured on the surface of allergen-stimulated basophils. BAT has been studied in the diagnosis of a variety of food allergies and its reported sensitivity ranges from 77% to 98%, and specificity from 75% to 100% [36,37,38,39,40,41].

The DBPCFC is the gold standard for the diagnosis of food allergies. It consists of gradually administering the suspect food under medical supervision in order to assess the clinical reaction or state of tolerance to said food. As the test is potentially dangerous, it must be carried out by qualified personnel in a suitable healthcare environment for managing any reactions. It is usually discontinued in the presence of a significant objective reaction or a persistent subjective reaction. In doubtful cases, it is repeated after a few days [31].

In daily clinical practice, the OFC is usually performed in open or single blind. The DBPCFC (in which both the patient and the clinicians do not know whether the “real” test food or a placebo is being eaten) is performed in doubtful cases or for research purposes. If the provocation test is negative, it is good practice to confirm it with an open test in which the patient eats the food in its natural form, as this helps exclude false negatives (which are around 1%–3%) [23,42].

The elimination diet is another very useful tool in the diagnosis of food allergy, especially when there is the onset of chronic skin or gastrointestinal reactions. This is normally used in the initial phase of diagnosis in order to verify the reduction of symptoms after exclusion for a few weeks. Elimination diets must always be carried out short-term and for a specific purpose [43].

The diagnosis of food allergies, with the exception of cases of IgE-mediated anaphylaxis, should, in fact, always be confirmed with OFC. Given that a state of tolerance towards the food to which one is allergic is often acquired over time, it is advisable to periodically (once a year) perform an in-vivo and in-vitro test of the individual allergic state, possibly also with an OFC [23].

Tests with no diagnostic value include the provocation–neutralization test, cytotoxic tests, kinesiology, and electrode dermal tests. In regards to the determination of specific IgG for food, their uselessness has been reported in diagnosing a food allergy or programming an exclusion diet [23]. Therefore, there are several tests to diagnose IgE-mediated food allergies; however, clinical history remains the most important tool to attain an accurate diagnosis. In vivo and in vitro tests can be used, together with clinical history, to reduce the need for OFC. The clinician’s task is to interpret the results of all available tests to decide if an OFC is needed to reach the diagnosis.

## 5. Prevention

Greater attention is being focused on the research of risk factors and preventive measures for reducing the risk of onset of food allergy. Important advances have been made over the last decades with the defining of three separate hypotheses on how food allergies develop, namely, the dual-allergen exposure hypothesis, the vitamin D hypothesis, and the hygiene hypothesis, which are summarized in Table 2 [44,45,46].

### Timing the Introduction of Complementary Foods and the Risk of Developing Food Allergies

An important and very heated discussion exists on the linking between the timing of the introduction of solid foods in infants and the risk of developing food allergies during various periods of life.

In the 1980s and 1990s, early exposure to solid foods (before four months of life) was associated with the development of allergic pathologies, especially eczema [47,48,49].

For this reason, a strategy of allergen avoidance in high-risk newborn infants (with a history of allergies in first-degree relatives) was thus adopted for many years, reducing—as much as possible—the condition of sensitized proteins, both in intrauterine life and during the first months of life (postponed introduction of potentially allergenic foods such as milk, eggs, fish, peanuts for 1–3 years of life) [50].

The avoidance strategy did not prove to be effective, given that in the following decades the incidence of allergic diseases and food allergies continued to increase.

All of this has led to abandoning the strategy of allergenic avoidance and, in fact, has induced several groups to carry out prospective intervention studies according to the hypothesis that the onset of food allergies in the general population, or in children with a high, medium, or low risk of developing food allergies (familiarity and/or atopic dermatitis and/or sensitization to food) could be prevented by the early introduction of common allergenic food [51,52,53,54,55,56,57,58,59].

With the change in evidence, the recommendations suggested by the most important international guidelines have also changed, as summarized in Table 3.

Solid foods should be introduced in the infant’s diet in accordance with familial and cultural habits, and when possible, while continuing breastfeeding up to two years of age or beyond [61,66]. Therefore, beginning from the fourth month of life, an infant should try almost all foods, according to his/her taste and acquired neuromotoric development stage, such as the ability to chew, to keep the head up, and to stay sitting down appropriately. In infants with food allergies and/or severe eczema [57] with positive SPT to a specific food, the food should be introduced into his/her diet after an oral food challenge conducted under medical supervision.

Understanding how to prevent the development of food allergies will expand knowledge to maintain tolerance to food antigens.

## 6. Management

Many authors consider that the only strategy currently available for the treatment of food allergies is the strict elimination diet. This type of attitude, which we could define as “passive”, does not overcome the risk of accidental reactions due to the involuntary intake of the culprit food. [67]. Patients with food allergies, at risk of serious reactions, should always carry a drug kit with an adrenaline autoinjector for immediate self-treatment [23]. For food allergy management, an “active” approach is urgently needed, such as specific allergen immunotherapy, which is currently being developed and only used for research purposes [67,68].

There are different types of immunotherapy, based on the administration route, and namely, oral immunotherapy (OIT) where the allergen is swallowed, sublingual immunotherapy (SLIT) where the allergen is held under the tongue for 2 min and then split or swallowed, and epicutaneous immunotherapy (EPIT), where a patch with a food allergen is applied on the skin [69].

**Figure 2:**
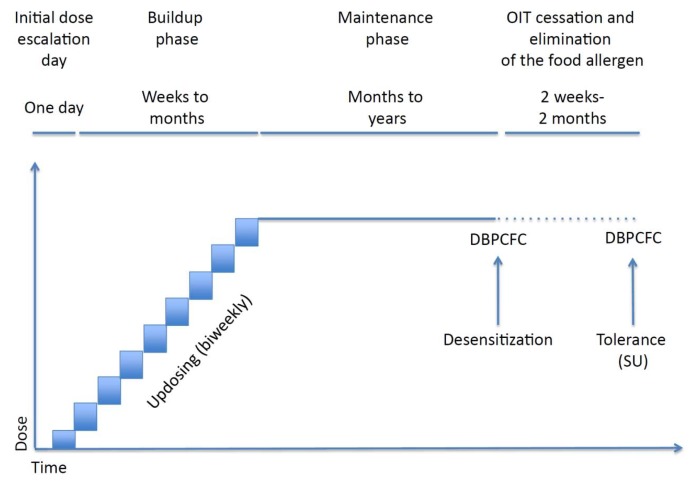
Typical protocol of food oral immunotherapy (OIT) (from Gernez Y. et al. [70]).

The typical protocol of oral immunotherapy includes an initial escalation phase followed by dose build-up and maintenance phases, Figure 2.

The initial escalation phase is often carried out on a single day and its purpose is to identify the starting daily dose for home administration.

During the build-up phase, the daily dose is normally increased every 15 days until the maintenance dose is reached. At home, the patients should continue to take the same maintenance dose every day. After some months/one year, a DBPCFC is performed to verify tolerance.

To assess a sustained unresponsiveness, the daily dose is then stopped for a period of 4 to 12 weeks and reintroduced during a DBPCFC. If no adverse reaction occurs, this state is defined permanent tolerance [70].

The aim of immunotherapy is to reach “a state of tolerance” in which the patient does not show any reaction after ingestion of a normal serving of the culprit food despite a period of absence of exposure. If the tolerance is not reached, we talk about desensitization, which indicates the ability to safely assume the culprit food, but it strictly depends on the daily intake of the same food [71].

The OIT induces desensitization (relative risk (RR) = 0.16 (95% CI 0.10–0.26)) but there is no evidence that oral immunotherapy induces long-term tolerance (RR = 0.29 (95% CI 0.08–1.13)) [69].

A study on egg oral immunotherapy conducted by Jones et al. [72] shows how the tolerance is enhanced with the duration of oral immunotherapy. The sustained unresponsiveness increases from 27.5% after 2 years of oral immunotherapy, up to 50% after 4 years.

While the SLIT induces desensitization (RR = 0.26 (95% CI 0.13–0.64)), it is not as good as OIT [69]. In fact, in a study on SLIT for peanut, conducted by Burks et al. [73], the sustained unresponsiveness was reached in only 11% of the patients.

Regarding safety, the risk of systemic reactions is higher in those receiving OIT compared to placebo (RR = 1.16, (95% CI 1.03; 1.30)). The local reactions are mild, such as oral allergic syndrome or abdominal pain (RR = 2.14, (95% CI 1.47; 3.12)) [69].

Eosinophilic esophagitis (EoE) is a long-term side effect of OIT, and its prevalence in subjects undergoing OIT varies from 2.7% [74] to 30% [75]. This wide variability derives from the fact that not all patients who develop gastrointestinal symptoms during OIT undergo a gastroesophageal biopsy; therefore, with the risk of overestimating this side effect.

A recent study [76] showed that OIT-induced EoE can be treated with a slower dosage regimen and a lower maintenance dose.

In SLIT, the systemic reactions are less frequent and milder, similar to a placebo arm (RR 0.98 (0.85–1.14)). The local reactions are frequent (7–40% of patients) and represented by the oral allergic syndrome [69]. To date, there have been no cases of EoE developing during the SLIT for the management of food allergy.

An alternative immunotherapy route, to improve the safety of OIT, is epicutaneous immunotherapy. It consists of a patch, called Viaskin, which is applied on the skin. The allergen protein, adhering to the inside of its surface, is dissolved by the moisture from natural trans-epidermal water loss accumulated under the patch. The permeability of the stratum corneum, increased by the moisture collected under the patch, allows native proteins to concentrate near antigen-presenting immune cells. The upper skin is not vascularized; therefore, the systemic absorption of the allergen is almost eliminated [77].

A study on peanuts demonstrated that patients treated with Viaskin did experience a significant increase in a successfully consumed dose compared to the placebo group (placebo vs. VP100 (Viaskin peanut 100 mcg), *p* = 0.014; placebo vs. VP250 (Viaskin peanut 250 mcg), *p* = 0.003); in particular the study showed that younger children experienced a more favorable outcome (*p* = 0.03; age, 4–11 vs. >11 years) [78].

EPIT seems to be safe and well tolerated. The most frequent reaction is a local skin reaction at the application site [78].

A preliminary study on EPIT with milk failed to demonstrate a statistically significant improvement of the cumulative tolerated dose between the active group and the placebo group [79]. The main characteristics of the three immunotherapy routes are summarized in Table 4.

Another way to improve safety is the use of processed food, especially baked egg and milk. Thermal processing alters allergenicity via denaturation of the epitopes, or by altering susceptibility to digestion. Allergenicity may be further reduced via interaction with wheat proteins, in particular gluten, affecting solubility and bioavailability [80].

In baked-food reactive patients, the OIT with baked products is considered as immunotherapy, whereas in baked-food, tolerant patients, it is instead a marker of a milder, more transient allergy phenotype [81]. In 2017, there was very weak evidence from a systematic review that baked egg and milk OIT could accelerate the acquisition of tolerance [82].

In 2018, a randomized controlled trial conducted on 84 patients with milk allergy demonstrated that a statistically significant higher percentage of patients who consumed baked products reached a tolerance to unheated milk versus patients not consuming baked products [83].

An anti-IgE monoclonal antibody was first proposed as an adjuvant to facilitate OIT by reducing allergic reactions induced by OIT [84].

There are studies on omalizumab and OIT with peanuts, [85,86] egg, [87] milk [88,89] and multiple foods [90]. All these studies demonstrate that omalizumab enables faster achievement of the target maintenance dose and reduces the rate and severity of IgE-mediated reactions during oral immunotherapy.

The use of omalizumab is off label for food allergy immunotherapy and its use is recommended for patients with severe food allergy who failed to be cured by oral immunotherapy [91].

A new therapeutic option, which showed promising results, is the use of probiotics with OIT. Tang et al. [92] has studied the co-administration of Lactobacillus rhamnosus with peanut oral immunotherapy in children with peanut allergies in a double-blind, placebo-controlled trial; 82.1% of patients in the active group compared to 3.6% of patients receiving placebo exhibited permanent tolerance within three weeks after stopping treatment.

A follow-up study four years after discontinuing treatment found that the sustained unresponsiveness was maintained by 7 out of 12 patients in the active group versus 1 out of 15 in the placebo group [93].

In terms of food allergy treatment strategies, it remains unsatisfactory that, at the moment, the only effective treatment is the elimination diet. In addition to immunotherapy, the use of biologics also appears to be promising. However, more and larger clinical trials are needed to clarify the potential of these therapeutic strategies.

## 7. Conclusions

In conclusion, more research on strategies for optimization of treatment is needed—such as combining administration routes to improve both efficacy and safety of immunotherapy. Moreover, the use of immunomodulatory agents is being developed and, depending on their results, they could become an important possible treatment for food allergies.

## Figures and Tables

**Table 1:** Predictive value of cut-offs for skin prick test (SPT) weal diameter and specific immunoglobulin E (s-IgE) level for a positive oral food challenge (OFC) (modified from Sampson H.A. et al. [25]).

Food	>95% PPV	
	SPT (mm)	s-IgE (KU/L)
Egg white	≥7	≥7
Cow’s milk	≥8	≥15
Peanut	≥8	≥14
Fish		≥20

PPV: positive predictive value.

**Table 2:** Integrating hypotheses of food allergy (modified from Renz H. et al. [2]).

Tolerance	Allergy	Ref.	Hypothesis
**Low rates of infant eczema:** High ambient humidity Limited use of harsh detergents	**High rates of infant eczema:** Frequent use of harsh detergents	[44]	Dual allergen exposure
**High level of vitamin D:** Increased UV light exposure Reduced sun avoidance	**Vitamin D insufficiency:** Sun avoidance Decreased UV light exposure	[45]	Vitamin D
**Dietary factors:** Boiled peanuts instead of roasted High intake of fruits and vegetables Non-Westernized feeding patterns	**Dietary factors:** Westernized diet Westernized infant feeding patterns	[44]	Dual allergen exposure
**High microbial exposure:** Large families, crowded living conditions Frequent and diverse exposure to animals (pets or livestock) Limited access to clean food and water High rates of H. pylori and parasitic infections Limited use of antibiotics in personal health and food chain Low rates of caesarean section delivery	**Low microbial exposure:** Small families Limited exposure to animals Limited food diversity	[46]	Hygiene

Ref: references.

**Table 3:** Recent recommendations for food introduction to prevent food allergies in the general population and in high-risk infants (modified from Caffarelli C. et al. [60]).

Scientific Society–Year (Reference)				
ASCIA, 2016 [61]	NIAID, 2017 [62]	ESPGHAN, 2017 [63]	APAPARI, 2018 [64]	BSACI 2018 [65]
Around 6 months, but not before 4 months. Peanut and cooked egg before 12 months. In infants with severe eczema, or egg allergy, or other food allergy, the modality of how to introduce peanuts into the diet should be discussed.	**Infants without eczema or food allergy** Peanuts should be introduced into the diet, according to the age as well as the preferences and cultural habits of the family. **Infants with mild–moderate eczema** Introduction of peanuts around 6 months of age, in accordance with family habits. **Infants with severe eczema and/or egg allergy** Introduction of peanuts at 4–6 months after performing s-IgE or SPT to peanuts. Infants with peanut s-IgE < 0.35 kUA/L and/or peanut SPT wheal of 2 mm or less, should introduce peanuts at home or in the office when there are parental concerns. Infants with peanut s-IgE > 0.35 kUA/L and/or peanut SPT wheal of 3–7 mm should perform supervised oral peanut challenge. Infants with peanut SPT wheal > 8 mm are probably allergic to peanuts. They should continue to be managed by a specialist.	Traditions and feeding patterns in the population on types of complementary foods should be considered. Complementary foods ≥ 4–6 months. Allergenic foods ≥ 4 months. Infants at high risk of peanut allergy (severe eczema, egg allergy, or both) Should introduce peanuts between 4 and 11 months; following evaluation by an appropriately trained professional.	**General population/at-risk infants (atopic family history, non-severe eczema)** Complementary foods (including allergenic foods) ≥ 6 months. Continue breastfeeding up to 2 years. **High risk infants with severe eczema** Allergy testing (skin prick tests and/or s-IgE to egg) (and peanuts in countries with high peanut allergy prevalence) should be preliminary performed, followed by a supervised oral challenge in sensitized children. In countries with limited access to allergy tests. Expertise-only supervised oral challenges to egg (and peanuts in countries with high peanut allergy prevalence) should be performed. **General population** Complementary foods (including allergenic foods) from around 6 months. Introduction of allergenic foods should not be delayed.	**General population** Complementary foods (including allergenic foods) from around 6 months. **High risk infants with eczema (particularly early-onset or moderate–severe eczema) or food allergy** Introduction of eggs and peanuts from 4 months. The benefits of allergy testing prior to introducing eggs and peanuts should be balanced against the risk of a delayed introduction.

APAPARI: Asian Pacific Association of Pediatric Allergy, Respirology and Immunology; ASCIA: Australasian Society of Clinical Immunology and Allergy; BSACI: British Society for Allergy and Clinical Immunology; ESPGHAN: European Society for Paediatric Gastroenterology, Hepatology, and Nutrition; NIAID: National Institute of Allergy and Infectious Diseases.

**Table 4:** Comparison of food allergen immunotherapies (modified from Gernez Y. et al. [70]).

	OIT	SLIT	EPIT
**Foods studied**	Peanut, milk, egg, wheat	Peanut, milk, hazelnut, peach	Peanut, milk
**Maintenance dose**	300–4000 mg	2–7 mg	50–500 μg (usually 250 μg)
**Efficacy**	More desirable Large effect on desensitisation	Less desirable Moderate effect	Currently being investigated
**Safety**	Less desirable	More desirable	More desirable
**Adverse effects**	Common during up-dosing Mostly gastrointestinal Can be systemic especially with co-factors; EoE < 8%	Mostly oro-pharyngeal Systemic reactions are rare	Local skin reactions
**Adherence**	Less good (especially due to GI symptoms)	Better than with OIT	Better than with OIT
**Feasibility**	Less good due to GI AE and changes to lifestyle	Easy	Easy

EoE: eosinophilic esophagitis; EPIT: epicutaneous immunotherapy; GI: gastrointestinal; OIT: oral immunotherapy; SLIT: sublingual immunotherapy; AE: adverse event.

## References

[1] Boyce J.A., Assa’ad A., Burks A.W., Jones S.M., Sampson H.A., Wood R.A., Plaut M., Cooper S.F., Fenton M.J., Arshad S.H. (2010). Guidelines for the diagnosis and management of food allergy in the United States: Summary of the NIAID-sponsored expert panel report. J. Allergy Clin. Immunol..

[2] Renz H., Allen K.J., Sicherer S.H., Sampson H.A., Lack G., Beyer K., Oettgen H.C. (2018). Food allergy. Nat. Rev. Dis. Primers.

[3] Nwaru B.I., Hickstein L., Panesar S.S., Roberts G., Muraro A., Sheikh A. (2014). EAACI Food Allergy and Anaphylaxis Guidelines Group. Prevalence of common food allergies in Europe: A systematic review and meta-analysis. Allergy.

[4] Gupta R.S., Springston E.E., Warrier M.R., Smith B., Kumar R., Pongracic J., Holl J.L. (2011). The prevalence, severity, and distribution of childhood food allergy in the United States. Pediatrics.

[5] Winberg A., West C.E., Strinnholm Å., Nordström L., Hedman L., Rönmark E. (2015). Assessment of Allergy to Milk, Egg, Cod, and Wheat in Swedish Schoolchildren: A Population Based Cohort Study. PLoS ONE.

[6] McGowan E.C., Matsui E.C., Peng R., Salo P.M., Zeldin D.C., Keet C.A. (2016). Racial/ethnic and socioeconomic differences in self-reported food allergy among food-sensitized children in National Health and Nutrition Examination Survey III. Ann. Allergy Asthma Immunol..

[7] Sicherer S.H., Sampson H.A. (2010). Food allergy. J. Allergy Clin. Immunol..

[8] Mills E.N., Mackie A.R., Burney P., Beyer K., Frewer L., Madsen C., Botjes E., Crevel R.W., van Ree R. (2007). The prevalence, cost and basis of food allergy across Europe. Allergy.

[9] Osborne N.J., Koplin J.J., Martin P.E., Gurrin L.C., Lowe A.J., Matheson M.C., Ponsonby A.L., Wake M., Tang M.L., Dharmage S.C. (2011). HealthNuts Investigators. Prevalence of challenge-proven IgE-mediated food allergy using population-based sampling and predetermined challenge criteria in infants. J. Allergy Clin. Immunol..

[10] Schoemaker A.A., Sprikkelman A.B., Grimshaw K.E., Roberts G., Grabenhenrich L., Rosenfeld L., Siegert S., Dubakiene R., Rudzeviciene O., Reche M. (2015). Incidence and natural history of challenge-proven cow’s milk allergy in European children--EuroPrevall birth cohort. Allergy.

[11] Xepapadaki P., Fiocchi A., Grabenhenrich L., Roberts G., Grimshaw K.E., Fiandor A., Larco J.I., Sigurdardottir S., Clausen M., Papadopoulos N.G. (2016). Incidence and natural history of hen’s egg allergy in the first 2 years of life-the EuroPrevall birth cohort study. Allergy.

[12] Peters R.L., Koplin J.J., Gurrin L.C., Dharmage S.C., Wake M., Ponsonby A.L., Tang M.L.K., Lowe A.J., Matheson M., Dwyer T. (2017). The prevalence of food allergy and other allergic diseases in early childhood in a population-based study: HealthNuts age 4-year follow-up. J. Allergy Clin. Immunol..

[13] McWilliam V., Koplin J., Lodge C., Tang M., Dharmage S., Allen K. (2015). The Prevalence of Tree Nut Allergy: A Systematic Review. Curr. Allergy Asthma Rep..

[14] Moonesinghe H., Mackenzie H., Venter C., Kilburn S., Turner P., Weir K., Dean T. (2016). Prevalence of fish and shellfish allergy: A systematic review. Ann. Allergy Asthma Immunol..

[15] Yu W., Freeland D.M.H., Nadeau K.C. (2016). Food allergy: Immune mechanisms, diagnosis and immunotherapy. Nat. Rev. Immunol..

[16] McDole J.R., Wheeler L.W., McDonald K.G., Wang B., Konjufca V., Knoop K.A., Newberry R.D., Miller M.J. (2012). Goblet cells deliver luminal antigen to CD103+ dendritic cells in the small intestine. Nature.

[17] Niess J.H., Brand S., Gu X., Landsman L., Jung S., McCormick B.A., Vyas J.M., Boes M., Ploegh H.L., Fox J.G. (2005). CX3CR1-mediated dendritic cell access to the intestinal lumen and bacterial clearance. Science.

[18] Schülke S. (2018). Induction of Interleukin-10 producing dendritic cells as a tool to suppress allergen-specific T helper 2 responses. Front. Immunol..

[19] Sheng J., Chen W., Zhu H.J. (2015). The immune suppressive function of transforming growth factor-β (TGF-β) in human diseases. Growth.

[20] Wambre E., Bajzik V., DeLong J.H., O’Brien K., Nguyen Q.A., Speake C., Gersuk V.H., DeBerg H.A., Whalen E., Ni C. (2017). A phenotypically and functionally distinct human T(H)2 cell subpopulation is associated with allergic disorders. Sci. Transl. Med..

[21] Zhu J. (2015). T helper 2 (Th2) cell differentiation, type 2 innate lymphoid cell (ILC2) development and regulation of interleukin-4 (IL-4) and IL-13 production. Cytokine.

[22] Iweala O.I., Burks A.W. (2016). Food Allergy: Our Evolving Understanding of Its Pathogenesis, Prevention, and Treatment. Curr. Allergy Asthma Rep..

[23] Muraro A., Werfel T., Hoffmann-Sommergruber K., Roberts G., Beyer K., Bindslev-Jensen C., Cardona V., Dubois A., duToit G., Eigenmann P. (2014). EAACI Food Allergy and Anaphylaxis Guidelines Group. EAACI food allergy and anaphylaxis guidelines: Diagnosis and management of food allergy. Allergy.

[24] Heinzerling L., Mari A., Bergmann K.C., Bresciani M., Burbach G., Darsow U., Durham S., Fokkens W., Gjomarkaj M., Haahtela T. (2013). The skin prick test—European standards. Clin. Transl. Allergy..

[25] Sampson H.A., Aceves S., Bock S.A., James J., Jones S., Lang D., Nadeau K., Nowak-Wegrzyn A., Oppenheimer J., Perry T.T. (2014). Food allergy: A practice parameter update-2014. J. Allergy Clin. Immunol..

[26] Roberts G., Lack G. (2005). Diagnosing peanut allergy with skin prick and specific IgE testing. J. Allergy Clin. Immunol..

[27] Sporik R., Hill D.J., Hosking C.S. (2000). Specificity of allergen skin testing in predicting positive open food challenges to milk, egg and peanut in children. Clin. Exp. Allergy.

[28] Sampson H.A. (2001). Utility of food-specific IgE concentrations in predicting symptomatic food allergy. J. Allergy Clin. Immunol..

[29] Knight A.K., Shreffler W.G., Sampson H.A., Sicherer S.H., Noone S., Mofidi S., Nowak-Wegrzyn A. (2006). Skin prick test to egg white provides additional diagnostic utility to serum egg white- specific IgE antibody concentration in children. J. Allergy Clin. Immunol..

[30] Sampson H.A., van Wijk R.G., Bindslev-Jensen C., Sicherer S., Teuber S.S., Burks A.W., Dubois A.E., Beyer K., Eigenmann P.A., Spergel J.M. (2012). Standardizing double-blind, placebo-controlled oral food challenges: American Academy of Allergy, Asthma & Immunology European Academy of Allergy and Clinical Immunology PRACTALL consensus report. J. Allergy Clin. Immunol..

[31] Gomes-Belo J., Hannachi F., Swan K., Santos A.F. (2018). Advances in Food Allergy Diagnosis. Curr. Pediatr. Rev..

[32] Costa J., Mafra I., Carrapatoso I., Oliveira M.B. (2016). Hazelnut allergens: Molecular characterization, detection, and clinical relevance. Crit. Rev. Food Sci. Nutr..

[33] Masthoff L.J., Mattsson L., Zuidmeer-Jongejan L., Lidholm J., Andersson K., Akkerdaas J.H., Versteeg S.A., Garino C., Meijer Y., Kentie P. (2013). Sensitization to Cor a 9 and Cor a 14 is highly specific for a hazelnut allergy with objective symptoms in Dutch children and adults. J. Allergy Clin. Immunol..

[34] Hoffmann H.J., Santos A.F., Mayorga C., Nopp A., Eberlein B., Ferrer M., Rouzaire P., Ebo D.G., Sabato V., Sanz M.L. (2015). The clinical utility of basophil activation testing in diagnosis and monitoring of allergic disease. Allergy.

[35] Santos A.F., Lack G. (2016). Basophil activation test: Food challenge in a test tube or specialist research tool?. Clin. Transl. Allergy.

[36] Glaumann S., Nopp A., Johansson S.G., Rudengren M., Borres M.P., Nilsson C. (2012). Basophil allergen threshold sensitivity, CD-sens, IgE- sensitization and DBPCFC in peanut-sensitized children. Allergy.

[37] Ocmant A., Mulier S., Hanssens L., Goldman M., Casimir G.L., Mascart F., Schandené L. (2009). Basophil activation tests for the diagnosis of food allergy in children. Clin. Exp. Allergy.

[38] Sato S., Tachimoto H., Shukuya A., Kurosaka N., Yanagida N., Utsunomiya T., Iguchi M., Komata T., Imai T., Tomikawa M. (2010). Basophil activation marker CD203c is useful in the diagnosis of hen’s egg and cow’s milk allergies in children. Int. Arch. Allergy Immunol..

[39] Tokuda R., Nagao M., Hiraguchi Y., Hosoki K., Matsuda T., Kouno K., Morita E., Fujisawa T. (2009). Antigen-induced expression of CD203c on basophils predicts IgE-mediated wheat allergy. Allergol. Int..

[40] Santos A.F., Douiri A., Becares N., Wu S.Y., Stephens A., Radulovic S., Chan S.M., Fox A.T., Du Toit G., Turcanu V. (2014). Basophil activation test discriminates between allergy and tolerance in peanut-sensitized children. J. Allergy Clin. Immunol..

[41] Gamboa P.M., Cáceres O., Antepara I., Sánchez-Monge R., Ahrazem O., Salcedo G., Barber D., Lombardero M., Sanz M.L. (2007). Two different profiles of peach allergy in the north of Spain. Allergy.

[42] Nowak-Wegrzyn A., Assa’ad A.H., Bahna S.L., Bock S.A., Sicherer S.H., Teuber S.S., Adverse Reactions to Food Committee of American Academy of Allergy, Asthma & Immunology (2009). Adverse Reactions to Food Committee of American Academy of Allergy, Asthma & Immunology. Work Group report: Oral food challenge testing. J. Allergy Clin. Immunol..

[43] Wood R.A. (2015). Diagnostic elimination diets and oral food provocation. Chem. Immunol. Allergy.

[44] Lack G. (2008). Epidemiologic risks for food allergy. J. Allergy Clin. Immunol..

[45] Weiss S., Litonjua A.A. (2008). Childhood asthma is a fat- soluble vitamin deficiency disease. Clin. Exp. Allergy.

[46] Strachan D.P. (1989). Hay fever, hygiene, and household size. BMJ.

[47] Fergusson D.M., Horwood L.J., Shannon F.T. (1983). Asthma and infant diet. Arch. Dis. Child..

[48] Kajosaari M., Saarinen U.M. (1983). Prophylaxis of atopic disease by six months’ total solid food elimination. Evaluation of 135 exclusively breast-fed infants of atopic families. Acta Paediatr. Scand..

[49] Forsyth J.S., Ogston S.A., Clark A., Florey C.D., Howie P.W. (1993). Relation between early introduction of solid food to infants and their weight and illnesses during the first two years of life. BMJ.

[50] Kleinman R.E. (2000). American Academy of Pediatrics recommendations for complementary feeding. Pediatrics.

[51] Du Toit G., Roberts G., Sayre P.H., Bahnson H.T., Radulovic S., Santos A.F., Brough H.A., Phippard D., Basting M., Feeney M. (2015). Randomized trial of peanut consumption in infants at risk for peanut allergy. N. Engl. J. Med..

[52] Du Toit G., Sayre P.H., Roberts G., Sever M.L., Lawson K., Bahnson H.T., Brough H.A., Santos A.F., Harris K.M., Radulovic S. (2016). Effect of avoidance on peanut allergy after early peanut consumption. N. Engl. J. Med..

[53] Palmer D.J., Metcalf J., Makrides M., Gold M.S., Quinn P., West C.E., Loh R., Prescott S.L. (2013). Early regular egg exposure in infants with eczema: A randomized controlled trial. J. Allergy Clin. Immunol..

[54] Palmer D.J., Sullivan T.R., Gold M.S., Prescott S.L., Makrides M. (2017). Randomized controlled trial of early regular egg intake to prevent egg allergy. J. Allergy Clin. Immunol..

[55] Bellach J., Schwarz V., Ahrens B., Trendelenburg V., Aksünger Ö., Kalb B., Niggemann B., Keil T., Beyer K. (2017). Randomized placebo- controlled trial of hen’s egg consumption for primary prevention in infants. J. Allergy Clin. Immunol..

[56] Wei-Liang Tan J., Valerio C., Barnes E.H., Turner P.J., Van Asperen P.A., Kakakios A.M., Campbell D.E., Beating Egg Allergy Trial (BEAT) Study Group (2017). A randomized trial of egg introduction from 4 months of age in infants at risk for egg allergy. J. Allergy Clin. Immunol..

[57] Natsume O., Kabashima S., Nakasato J., Yamamoto-Hanada K., Narita M., Kondo M., Saito M., Kishino A., Takimoto T., Inoue E. (2017). Two-step egg introduction for prevention of egg allergy in high-risk infants with eczema (PETIT): A randomised, double-blind, placebo-controlled trial. Lancet.

[58] Perkin M.R., Logan K., Tseng A., Raji B., Ayis S., Peacock J., Brough H., Marrs T., Radulovic S., Craven J. (2016). Randomized trial of intro- duction of allergenic foods in breast fed infants. N. Engl. J. Med..

[59] Grimshaw K., Logan K., O’Donovan S., Kiely M., Patient K., van Bilsen J., Beyer K., Campbell D.E., Garcia-Larsen V., Grabenhenrich L. (2017). Modifying the infant’s diet to prevent food allergy. Arch. Dis. Child..

[60] Caffarelli C., Di Mauro D., Mastrorilli C., Bottau P., Cipriani F., Ricci G. (2018). Solid Food Introduction and the Development of Food Allergies. Nutrients.

[61] Campbell D., Vale S., Smith J., Roche I., Netting M., Allen K. (2016). ASCIA-P5: ASCIA Guidelines for Infant Feeding and Allergy Prevention. Intern. Med. J..

[62] Togias A., Cooper S.F., Acebal M.L., Assa’ad A., Baker J.R., Beck L.A., Block J., Byrd-Bredbenner C., Chan E.S., Eichenfield L.F. (2017). Addendum guidelines for the prevention of peanut allergy in the United States: Report of the National Institute of Allergy and Infectious Diseases-sponsored expert panel. J. Allergy Clin. Immunol..

[63] Fewtrell M., Bronsky J., Campoy C., Domellöf M., Embleton N., Mis N.F., Hojsak I., Hulst J.M., Indrio F., Lapillonne A. (2017). Complementary feeding: A position paper by the European Society for Paediatric Gastroenterology, Hepatology, and Nutrition (ESPGHAN) Committee on Nutrition. J. Pediatr. Gastroenterol. Nutr..

[64] Tham E.H., Shek L.P.-C., Van Bever H.P., Vichyanond P., Ebisawa M., Wong G.W., Lee B.W. (2018). Asia Pacific Association of Pediatric Allergy, Respirology & Immunology (APAPARI). Early introduction of allergenic foods for the prevention of food allergy from an Asian perspective—An Asia Pacific Association of Pediatric Allergy, Respirology & Immunology (APAPARI) consensus statement. Pediatr. Allergy Immunol..

[65] British Society for Allergy and Clinical Immunology/British Dietetic Association (2018). Preventing Food Allergy in Higher Risk Infants: Guidance for Healthcare Professionals. https://www.bsaci.org/about/early-feeding-guidance.

[66] World Health Organization (2010). Nutrition: Exclusive Breastfeeding. http://www.who.int/nutrition/topics/exclusive_breastfeeding/en/index.html.

[67] Worm M., Reese I., Ballmer-Weber B., Beyer K., Bischoff S.C., Classen M., Fischer P.J., Fuchs T., Huttegger I., Jappe U. (2015). Guidelines on the management of IgE mediated food allergies. Guidelines of the German Society for Allergology and Clinical Immunology. Allergo J. Int..

[68] Narisety S.D., Frischmeyer-Guerrerio P.A., Keet C.A., Gorelik M., Schroeder J., Hamilton R.G., Wood R.A. (2015). A randomized, double-blind, placebo-controlled pilot study of sublingual versus oral immunotherapy for the treatment of peanut allergy. J. Allergy Clin. Immunol..

[69] Nurmatov U., Dhami S., Arasi S., Pajno G.B., Fernandez-Rivas M., Muraro A., Roberts G., Akdis C., Alvaro-Lozano M., Beyer K. (2017). Allergen immunotherapy for IgE-mediated food allergy: A systematic review and meta-analysis. Allergy.

[70] Gernez Y., Nowak-Węgrzyn A. (2017). Immunotherapy for Food Allergy: Are We There Yet?. J. Allergy Clin. Immunol. Pract..

[71] Pajno G.B., Castagnoli R., Muraro A., Alvaro-Lozano M., Akdis C.A., Akdis M., Arasi S. (2019). Allergen immunotherapy for IgE-mediated food allergy: There is a measure in everything to a proper proportion of therapy. Pediatr. Allergy Immunol..

[72] Jones S.M., Burks A.W., Keet C., Vickery B.P., Scurlock A.M., Wood R.A., Liu A.H., Sicherer S.H., Henning A.K., Lindblad R.W. (2016). Consortium of Food Allergy Research (CoFAR). Long-term treatment with egg oral immunotherapy enhances sustained unresponsiveness that persists after cessation of therapy. J. Allergy Clin. Immunol..

[73] Burks A.W., Wood R.A., Jones S.M., Sicherer S.H., Fleischer D.M., Scurlock A.M., Vickery B.P., Liu A.H., Henning A.K., Lindblad R. (2015). Consortium of Food Allergy Research. Sublingual immunotherapy for peanut allergy: Long-term follow-up of a randomized multicenter trial. J. Allergy Clin. Immunol..

[74] Lucendo A.J., Arias A., Tenias J.M. (2014). Relation between eosinophilic esophagitis and oral immunotherapy for food allergy: A systematic review with meta-analysis. Ann. Allergy Asthma Immunol..

[75] Petroni D., Spergel J.M. (2018). Eosinophilic esophagitis and symptoms possibly related to eosinophilic esophagitis in oral immunotherapy. Ann. Allergy Asthma Immunol..

[76] Goldberg M.R., Nachshon L., Levy M.B., Elizur A., Katz Y. (2020). Risk Factors and Treatment Outcomes for Oral Immunotherapy-Induced Gastrointestinal Symptoms and Eosinophilic Responses (OITIGER). J. Allergy Clin. Immunol. Pract..

[77] Viaskin Epicutaneous Immunotherapy. https://www.dbv-technologies.com/viaskin-platform.

[78] Jones S.M., Sicherer S.H., Burks A.W., Leung D.Y., Lindblad R.W., Dawson P., Henning A.K., Berin M.C., Chiang D., Vickery B.P. (2017). Consortium of Food Allergy Research. Epicutaneous immunotherapy for the treatment of peanut allergy in children and young adults. J. Allergy Clin. Immunol..

[79] Dupont C., Kalach N., Soulaines P., Legoué-Morillon S., Piloquet H., Benhamou P.H. (2010). Cow’s milk epicutaneous immunotherapy in children: A pilot trial of safety, acceptability, and impact on allergic reactivity. J. Allergy Clin. Immunol..

[80] Vazquez-Ortiz M., Turner P.J. (2016). Improving the safety of oral immunotherapy for food allergy. Pediatr. Allergy Immunol..

[81] Nowak-Węgrzyn A., Lawson K., Masilamani M., Kattan J., Bahnson H.T., Sampson H.A. (2018). Increased Tolerance to Less Extensively Heat-Denatured (Baked) Milk Products in Milk-Allergic Children. J. Allergy Clin. Immunol. Pract..

[82] Lambert R., Grimshaw K.E.C., Ellis B., Jaitly J., Roberts G. (2017). Evidence that eating baked egg or milk influences egg or milk allergy resolution: A systematic review. Clin. Exp. Allergy.

[83] Esmaeilzadeh H., Alyasin S., Haghighat M., Nabavizadeh H., Esmaeilzadeh E., Mosavat F. (2018). The effect of baked milk on accelerating unheated cow’s milk tolerance: A control randomized clinical trial. Pediatr. Allergy Immunol..

[84] Lin C., Lee I.T., Sampath V., Dinakar C., DeKruyff R.H., Schneider L.C., Nadeau K.C. (2017). Combining anti-IgE with oral immunotherapy. Pediatr. Allergy Immunol..

[85] Schneider L.C., Rachid R., LeBovidge J., Blood E., Mittal M., Umetsu D.T. (2013). A pilot study of omalizumab to facilitate rapid oral desensitization in high-risk peanut-allergic patients. J. Allergy Clin. Immunol..

[86] Bock S.A., Sampson H.A., Atkins F.M., Zeiger R.S., Lehrer S., Sachs M., Bush R.K., Metcalfe D.D. (1988). Double-blind, placebo-controlled food challenge (DBPCFC) as an office procedure: A manual. J. Allergy Clin. Immunol..

[87] Lafuente I., Mazon A., Nieto M., Uixera S., Pina R., Nieto A. (2014). Possible recurrence of symptoms after discontinuation of omalizumab in anti-IgE-assisted desensitization to egg. Pediatr. Allergy Immunol..

[88] Wood R.A., Kim J.S., Lindblad R., Nadeau K., Henning A.K., Dawson P., Plaut M., Sampson H.A. (2016). A randomized, double-blind, placebo-controlled study of omalizumab combined with oral immunotherapy for the treatment of cow’s milk allergy. J. Allergy Clin. Immunol..

[89] Nadeau K.C., Schneider L.C., Hoyte L., Borras I., Umetsu D.T. (2011). Rapid oral desensitization in combination with omalizumab therapy in patients with cow’s milk allergy. J. Allergy Clin. Immunol..

[90] Begin P., Dominguez T., Wilson S.P., Bacal L., Mehrotra A., Kausch B., Trela A., Tavassoli M., Hoyte E., O’Riordan G. (2014). Phase 1 results of safety and tolerability in a rush oral immunotherapy protocol to multiple foods using Omalizumab. Allergy Asthma Clin. Immunol..

[91] El-Qutob D. (2016). Off-Label Uses of Omalizumab. Clin. Rev. Allergy Immunol..

[92] Tang M.L., Ponsonby A.L., Orsini F., Tey D., Robinson M., Su E.L., Licciardi P., Burks W., Donath S. (2015). Administration of a probiotic with peanut oral immunotherapy: A randomized trial. J. Allergy Clin. Immunol..

[93] Hsiao K.-C., Ponsonby A.-L., Axelrad C., Pitkin S., Tang M.L.K. (2017). Long-term clinical and immunological effects of probiotic and peanut oral immunotherapy after treatment cessation: 4-year follow-up of a randomised, double-blind, placebo-controlled trial. Lancet Child Adolesc. Health.

